# Sex differences and effects of the estrous stage on hippocampal‐prefrontal theta communications

**DOI:** 10.14814/phy2.14646

**Published:** 2020-11-23

**Authors:** Kristin J. Schoepfer, Yiqi Xu, Aaron A. Wilber, Wei Wu, Mohamed Kabbaj

**Affiliations:** ^1^ Department of Biomedical Sciences Florida State University College of Medicine Tallahassee FL USA; ^2^ Department of Statistics Florida State University Tallahassee FL USA; ^3^ Department of Psychology Florida State University Tallahassee FL USA

**Keywords:** estrous, sex differences, theta oscillations

## Abstract

Effective communication between the mammalian hippocampus and neocortex is essential to certain cognitive‐behavioral tasks critical to survival in a changing environment. Notably, functional synchrony between local field potentials (LFPs) of the ventral hippocampus (vHPC) and the medial prefrontal cortex (mPFC) within the theta band (4–12 Hz) underlies innate avoidance behavior during approach‐avoidance conflict tasks in male rodents. However, the physiology of vHPC‐mPFC communications in females remains unestablished. Furthermore, little is known about how mPFC subdivisions functionally interact in the theta band with hippocampal subdivisions in both sexes in the absence of task demand. Given the established roles of biological sex and gonadal hormone status on innate avoidance behaviors and neuronal excitability, here, we characterize the effects of biological sex and female estrous stage on hippocampal‐prefrontal (HPC‐mPFC) theta signaling in freely moving female and male rats. LFPs from vHPC, dorsal hippocampus (dHPC), mPFC‐prelimbic (PrL), and mPFC‐infralimbic (IL) were simultaneously recorded during spontaneous exploration of a familiar arena. Data suggest that theta phase and power in vHPC preferentially synchronize with PrL; conversely, dHPC and IL preferentially synchronize. Males displayed greater vHPC‐PrL theta synchrony than females, despite similar regional frequency band power and inter‐regional coherence. Additionally, several significant estrous‐linked changes in HPC‐mPFC theta dynamics were observed. These findings support the hypothesis that HPC‐mPFC theta signaling is sensitive to both biological sex and female estrous stage. These findings establish novel research avenues concerning sex as a biological variable and effects of gonadal hormone status on HPC‐mPFC network activity as it pertains to threat evaluation biomarkers.

## Graphical abstract

Effective signaling between subregions of the hippocampus and the prefrontal cortex underlies several cognitive‐behavioral processes necessary for survival. Theta‐frequency synchrony between the local fields of the ventral hippocampus and the prefrontal cortex regulates innate avoidance behavior in male rodents. However, this circuit remains understudied in females, despite its potential utility in research modeling the neurobiology of anxiety disorders (and sex differences therein). Here, we demonstrate that both biological sex and female hormone status have multifaceted effects on hippocampal‐prefrontal theta‐frequency signaling in freely‐moving rats during the exploration of a familiar arena. These findings provide insight into how biological sex and the female estrous stage shape the way in which these circuits may represent the potential for threat.

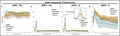

## 1 INTRODUCTION

The brain's ability to adaptively respond to the body's surroundings relies on synchronous oscillatory activity in the local field potentials (LFPs) of interconnected brain regions (Buzsáki, 2009). In mammals, LFP interactions between subregions of the hippocampus (HPC) and the medial prefrontal cortex (mPFC) play a critical role in many cognitive functions critical for survival, for example, encoding and retrieving environmental cue information associated with a goal‐directed behavior (Colgin, 2011; Harris & Gordon, 2015). Indeed, disrupted hippocampal‐prefrontal (HPC‐mPFC) interactions have been linked with several psychiatric disease states and may contribute to their pathophysiology (Sigurdsson & Duvarci, 2016). Along the dorsal‐ventral (septal‐temporal) axis of the hippocampus, multiple overlapping gradients of gene expression and anatomical connectivity are believed to produce distinct functional processes and behavioral outputs (Fanselow & Dong, 2010; Strange et al., 2014). In rats, the ventral hippocampus CA1 (vHPC) sends bilateral, long‐range, glutamatergic projections directly to ipsilateral dorsal subregions of the mPFC: namely, the prelimbic cortex (PrL) and infralimbic cortex (IL) (Hoover & Vertes, 2007; Jay & Witter, 1991; Thierry et al., 2000). Similarly, in humans, the hippocampus is connected to regions of the medial prefrontal cortex by white matter tracts(Teipel et al., 2010). Unlike the vHPC, neurons in the rat dorsal hippocampus CA1 (dHPC) do not directly project to the mPFC, instead primarily relaying first in midline nuclei of the thalamus (Vertes, 2006).

Synchronized functional connectivity in the vHPC‐mPFC circuit is believed to underlie the processing of environmental and subjective cues in order to assign emotional valence to the environment and guide an appropriate behavioral response based on the perceived threat (Calhoon & Tye, 2015). Several findings in male mice have demonstrated that theta frequency (~8 Hz) phase synchrony in vHPC‐mPFC LFPs is critical to the expression of innate avoidance behavior in approach‐avoidance conflict tasks such as the elevated plus maze (Adhikari et al., 2010; Padilla‐Coreano et al., 2016, 2019). Optogenetic experiments have revealed that the vHPC‐mPFC pathway is necessary for typical avoidance behavioral expression, theta power correlations between the regions, and for synchronizing mPFC single units to vHPC theta (Padilla‐Coreano et al., 2016), whereas inducing sinusoidal theta‐frequency oscillations from vHPC to mPFC is sufficient to generate overly avoidant behaviors and to enhance the phase‐locking of mPFC units to vHPC theta while in the aversive zones of the maze (Padilla‐Coreano et al., 2019). These findings have direct relevance to understanding the neurobiology of innate threat processing, and are of value in modeling “anxiety‐like” states in rodents, as homologous brain regions to the rodent HPC and mPFC are involved in human expression of anxiety states and recall of extinguished fear (Bach et al., 2014; Blair et al., 2011; Boehme et al., 2013; Milad et al., 2007).

Anxiety is a naturally occurring adaptive response to a potential threat. However, when overexpressed in inappropriate contexts, it can result in an anxiety disorder: a class of psychiatric disease that disproportionately affects women (McLean et al., 2011). Sex differences in mammalian behaviors are due, in part, to the developmental and activational effects of circulating gonadal steroid hormones on the structure, and therefore the function, of neurons and other cells in the central nervous system (McEwen, 1981). Along with other interconnected limbic structures including the amygdala, neurons in the HPC and mPFC are rich with receptors for androgens, estrogens, and progesterone (Guerra‐Araiza et al., 2002; Simerly et al., 1990). Furthermore, natural cycling of estradiol and progesterone dynamically alters innate avoidance (“anxiety‐like”) behaviors in female rodents (Frye et al., 2000; Marcondes et al., 2001; Mora et al., 1997) as well as functional connectivity between the HPC and mPFC in women (Arélin et al., (2015)). Together, this suggests a potential role for the hormonal milieu to act upon vHPC‐mPFC circuits or networks in rodents to modify emotional reactivity to potential threat and alter the expression of innate avoidance behaviors. Surprisingly, however, the degree to which biological sex and/or female hormonal status may affect the coherent activity within HPC‐mPFC networks has not yet been identified.

Here, we describe the roles of biological sex and female estrous stage on the neural oscillations within and between the mPFC and HPC of awake freely moving rats during active exploration of a familiar arena. Also, we compare functional connectivity patterns between subregions of the mPFC (PrL and IL) and subregions of the hippocampus (dorsal and ventral CA1).

## 2 MATERIALS AND METHODS

### 2.1 Animal subjects

Adult male (*n* = 4) and female (*n* = 5) Sprague Dawley rats aged 8 weeks obtained from Charles River Laboratories (Raleigh, NC) were used in this study. Animals were housed in same‐sex pairs with environmental enrichment for 3+ days before surgery and were singly housed with environmental enrichment after surgery. Simple environmental enrichment consisted of a PVC tube and a chew bone. Animals were kept under standard controlled temperatures, reversed 12‐hr light‐dark cycles (lights off at 10:00 a.m.), and were allowed ad libitum access to standard chow and water. All procedures were carried out under strict accordance with the NIH *Guide for the Care and Use of Laboratory Animals*, and the animal protocol was approved by the Florida State University Institutional Animal Care and Use Committee.

### 2.2 Electrode construction

Formvar‐insulated nickel‐chromium alloy wire (California Fine Wire, 0.003 in) was used to construct custom electrode probes. Six‐electrode bundles were constructed by twisting wire and adhering with a heat gun. The brain‐oriented bundle end was cut with a fresh scalpel at an angle such that the uninsulated tips of the six wires covered a 1 mm spread. Four‐electrode bundles were constructed similarly but with 1 mm spacing between each wire (total spread = 3 mm), and adhering them with glue away from the tips. The free ends of all bundles were fire‐stripped of insulation and were mechanically coupled with gold pin receptacles (Mill‐Max, #0489‐0‐15‐15‐11‐27‐04‐0) using TYGON ND‐100‐80 tubing (0.01 in ID; 0.03 in OD). A short piece of insulated wire serving as a reference electrode was coupled with a pin receptacle. Electrode impedances were tested in saline (nanoZ, White Matter, 1,004 Hz, 40 cycles); only bundles with impedances of 0.1–0.5 MΩ were used. Channels were line‐labeled in their bundle by passing current (−2 μA) into each wire in saline and mapping the resultant air bubble. An insulated micro coaxial ground wire (42 AWG, Molex #100065‐0023) was soldered to a gold pin receptacle and a stainless‐steel skull screw (Fine Science Tools, #19010‐00).

### 2.3 Surgery

Rats were deeply anesthetized with inhaled isoflurane and secured in a stereotaxic apparatus (Kopf Instruments). The skull angle was leveled (<50 μm) using bregma/lambda landmarks, 1–2 skull screws were manually driven into each bone plate, and the ground‐coupled screw was implanted in the left frontal bone plate near the olfactory bulb. Craniotomies were made on the right hemisphere over the implant coordinates, and durotomies were performed. Electrode bundles were lowered into the brain with a micromanipulator targeting the following coordinates: mPFC: AP + 3.0, ML + 0.7, DV −4.0 (4 channels); dHPC CA1: AP −3.8, ML + 2.5, DV −3.0 (6 channels); vHPC CA1: AP −5.5, ML + 5.0, DV –6.6 (6 channels; Paxinos & Watson, 2006). A reference wire was implanted in shallow cerebellar white matter (AP −12.08, ML + 0.85, DV −0.3). Anterior/posterior (AP) and medial/lateral (ML) coordinates are in reference to bregma, and dorsal/ventral (DV) coordinates are in reference to dura mater. Electrodes were affixed to the skull using C&B Metabond (Covetrus), then dental cement (Stoelting, #51458). The free ends of the electrodes were pinned out to a Mill‐Max header cut to 9×2 size (Mill‐Max, #853‐93‐100‐10‐001000), and all hardware was encased in dental cement, leaving the header ports open. Animals were monitored until emergence from anesthesia and were treated with analgesics and topical antibiotics as needed.

### 2.4 Behavioral protocol

Animals were allowed to recover for at least 10 days or until regaining pre‐surgery body weight and displaying a healthy scalp surrounding the implant. The recording arena consisted of a 46 × 46 × 55 cm gray acrylic box with an open top (Maze Engineers). Rats were acclimated to the recording arena, handling, and tethering to the headstage for three 10‐min sessions daily for 3 days. Animals were transported in light‐protected conditions to the dark recording room and were allowed to acclimate for 15 min. Staticide (ACL, Inc.) was applied to the arena's floor and walls before each trial. LFPs from PrL, IL, vHPC, and dHPC were recorded for 10 min in the recording chamber (“familiar arena”). All recordings occurred between +3–5 hr into the dark cycle and were performed in the dark. The arena was cleaned with 70% ethanol after each trial. Each animal provided 23–28 recordings (Table 1).

**Table 1:** Sample sizes. Biological and technical replicates included in this study

Subjects	FEMALE					MALE			
	“F1”	“F2”	“F3”	“F4”	“F5”	“M1”	“M2”	“M3”	“M4”
*n(Trials)*	25	28	23	26	28	27	27	27	25
*n(Trials.Diestrus)*	13	14	14	13	12				
*n(Trials.Proestrus)*	3	9	3	7	6				
*n(Trials.Estrus)*	6	2	5	5	9				
*n(Trials.Metestrus)*	3	3	1	1	1				

### 2.5 Data acquisition

Local field potentials (LFPs) from freely moving rats were recorded using a 16‐channel unity‐gain acquisition system (Neuralynx, Digital Lynx 4SX) at 32 kHz sampling rate, referenced to the ground screw, and band‐pass filtered 0.5–600 Hz. A monochrome video camera mounted overhead tracked an infrared LED mounted to the headstage preamplifier (XY coordinates) and captured video tracking data at 30 Hz.

### 2.6 Estrous stage determination

Vaginal epithelial cells were collected daily via sterile saline lavage from female rats immediately after each LFP recording (~3 hr into the dark cycle). Samples were visualized under a 10× brightfield microscope; cell cytology informed estrous stage (Becker et al., 2005). To account for potential stress associated with the lavage technique, males were handled similarly daily.

### 2.7 Data analysis

Data were imported into MATLAB (R2018b, MathWorks) and were analyzed with custom‐written scripts, inbuilt functions, functions from the Communication Toolbox and Signal Processing Toolbox, and open‐source packages. Data analysis scripts can be accessed on GitHub (https://github.com/KrisNeuro/KJS‐Thesis). LFPs were high‐pass filtered at 0.5 Hz, 60 Hz power line interference and six harmonics were attenuated (Keshtkaran & Yang, 2014), and LFPs were down‐sampled to 2 kHz. Individual channels were excluded from further analysis if found to be poorly grounded or of significantly lower amplitude than other channels in that brain region across all trials. Of the remaining channels, for each trial, a matrix of phase‐locking values (PLV) between 0.5 and 100 Hz was generated via the multitaper method (Chronux toolbox, http://chronux.org/; Mitra & Bokil, 2009) to compare all channels to each other. The mean PLV matrix was calculated, and channels within each brain region were visually inspected. Values ranged from 0 to 1; individual channels with PLV < 0.6 versus all other channels in that brain region were considered outliers and were excluded from further analysis. In each brain region, the mean voltage of remaining usable channels was calculated at each time point, generating a precleaned “regional LFP.” XY coordinates from the headstage LED tracker were upsampled with padding to 2 kHz, co‐registered with LFP timestamps, and converted to cm units. Animal linear movement velocities were calculated using the Freely Moving Animal Toolbox (http://fmatoolbox.sourceforge.net) smoothed with a Gaussian kernel (*SD* = 1 s). In freely moving rodents, hippocampal theta activity increases with running speed (McFarland et al., 1975). To reduce this variability, LFPs were velocity‐filtered to exclude periods of movement slower than 5 cm/s or faster than 15 cm/s in analyses of power spectral density, frequency band power, and coherence. For each subject, epochs of movement 5–15 cm/s from all familiar arena recordings were concatenated into a single long time series containing four simultaneously recorded pre‐cleaned LFPs from PrL, IL, vHPC, and dHPC. Power spectral densities were calculated using the Welch method (moving window of 0.4 s, 90% overlap between windows, 4,000 nFFTs). Plotted power spectral data are decibel‐transformed. For HPC‐mPFC coherence, 5–15 cm/s LFP data from each subject were concatenated, and magnitude‐squared coherence was calculated for each circuit between 0.5 and 100 Hz, then separately for the theta band (4–12 Hz) (2 s Hann window, 50% overlap). To calculate power correlations between areas, theta power was calculated for each brain region over time from the full trials, regardless of running velocity (multitaper cross‐spectrogram, 2.5 s window, no overlap between windows, NW = 2.5, 2048 nFFTs). Then, two‐tailed linear correlation coefficients (*r* ^2^) were calculated from each HPC‐mPFC pair for each trial. To calculate theta phase lags, LFPs (all movement velocities included) were band‐pass filtered for the theta band (4–12 Hz, Butterworth, MATLAB function “filtfilt” to correct for phase) To ensure accurate theta phase, data included only epochs when dHPC theta power was greater than its mean for that recording. Hilbert transform was applied to the theta‐filtered time series to extract instantaneous theta phase angles from each region. Values <−π or >π were adjusted to account for the oscillation wraparound. Instantaneous theta phase was subtracted between pairs of signals for each recording, and distributions of phase differences were analyzed, with values between −π and π in increments of π/100. Data were analyzed by calculating the width (in radians) of half the maximum phase lag distribution peak, such that tightly coupled theta oscillations have a narrow peak width and poorly coupled oscillations have a large peak width.

### 2.8 Statistical analyses

Data were analyzed using MATLAB or Prism (8.1.3, GraphPad) software. Two‐dimensional data (PSD, coherence) were analyzed using functional *F* tests and functional ANOVAs to compare the function (shape) of the curves. Here, all trial epochs of 5–15 cm/s movement were concatenated by the subject before calculating PSD or coherence, capturing all spectral data for that subject. For theta band power correlations and theta phase lags, comparisons between the sexes and between estrous stages were analyzed using a hierarchical bootstrapping approach adapted from Saravanan et al. (Saravanan et al., 2019). Trials were bootstrapped to the subject, resampling data 10^4^ permutations with replacement. Bootstrapped samples were statistically compared via the joint‐probability matrix, directly assessing the probability of the second variable being equal to or greater than the first (p_boot_). One‐tailed p_boot_ values were converted to two‐tailed *p* values via: 2*min(p_boot_, 1‐p_boot_). To compare global differences in theta band power correlations and theta phase lags between HPC‐mPFC circuits, subject means were analyzed via repeated‐measures one‐way ANOVA with Tukey's post hoc tests on normally distributed data, or Friedman test with Dunn's post hoc tests on abnormally distributed data. Alpha (*p*) was set to 0.05 when comparing independent data (males versus females). Significant *p* values were set to .0167 in estrous stage comparisons to account for three pairwise comparisons (Proestrus/Diestrus, Estrus/Diestrus, Estrus/Proestrus; or any estrous group versus males). Metestrus trials were excluded in analyses of estrous stage effects due to the small sample size. Statistical outputs and p values are reported in the figure legends.

### 2.9 Histology

After all recordings, animals were overdosed with sodium pentobarbital solution (Covetrus, succumb, delivered i.p.) and were transcardially perfused with paraformaldehyde (4% w/v in 100 mM phosphate‐buffered saline, pH 7.4). Brain tissue was collected and post‐fixed in 4% paraformaldehyde overnight at 4°C. Coronal brain tissue slices (40 μm) were collected using a vibratome (Leica VT1000S). Tissue was mounted on positively charged glass slides and was subject to Nissl staining with cresyl violet as described in Paul *et al*. (Paul et al., 2008). Electrode lesions were photographed using a 5× brightfield microscope and digital camera (Thermo Fisher, EVOS XL Core). Rats with inaccurate targeting were eliminated from the study.

## 3 RESULTS

**Figure 1:**
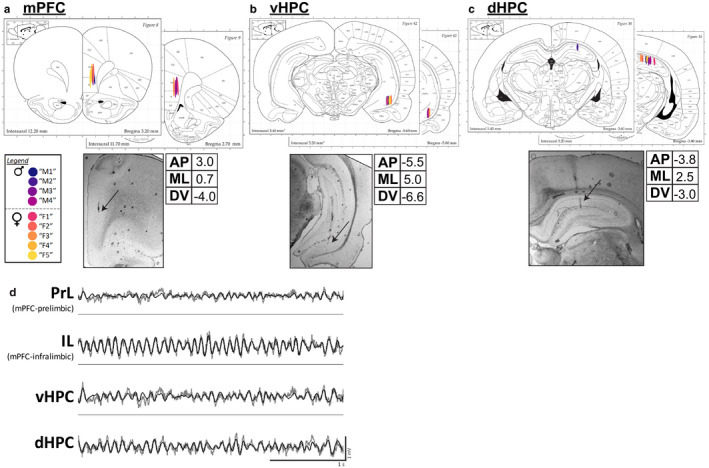
Electrode implant sites and representative LFP traces. (a) Medial prefrontal cortex (mPFC). Four‐electrode bundles with a total vertical spread of 3mm were implanted targeting mPFC‐Infralimbic (IL), such that the two dorsal‐most channels targeted mPFC‐Prelimbic (PrL). (b) Ventral hippocampus (vHPC). Six‐electrode bundles with a total vertical spread of 1mm were implanted targeting vHPC CA1. (c) Dorsal hippocampus (dHPC). Six‐electrode bundles with a total vertical spread of 1mm were implanted targeting dHPC CA1. Male and female implant locations are matched. Tables list stereotaxic coordinates (AP: anterior‐posterior; ML: medial‐lateral; DV: dorsal‐ventral).*Below*: Example of photographs of electrode lesion sites, 5× objective. Black arrows indicate tissue lesions. Brain atlas images are adapted from Paxinos and Watson (Paxinos &amp; Watson, 2006). (d) Representative traces of simultaneously‐recorded LFPs from PrL, IL, vHPC, and dHPC during active exploration in a familiar arena. Raw traces are plotted in gray and theta‐filtered traces are overlaid in black

Adult male and female rats were chronically implanted with electrode bundles targeting mPFC‐infralimbic (IL), mPFC‐prelimbic (PrL) (Figure 1a), ventral hippocampus CA1 (vHPC) (Figure 1b), and dorsal hippocampus CA1 (dHPC) (Figure 1c). Male and female implant locations were matched. After recovery, LFPs from the four brain regions were simultaneously recorded daily for 10‐min trials in a familiar arena during freely moving exploratory behavior (Figure 1d).

### 3.1 No differences in regional power spectra between sexes or estrous stages

**Figure 2:**
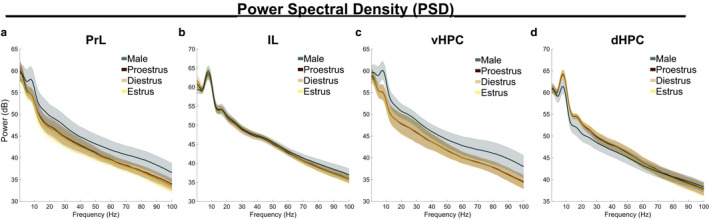
Power spectral density estimates in prefrontal and hippocampal subregions are similar between the sexes and across estrous stages. The shape of PSD estimates (0.5–100 Hz) did not statistically differ between the sexes or between females estrous stages in (a) PrL (sex*F* = 1.4291,*p* = .2745; estrous*F* = 6.6482 × 10^12^,*p* = .55955), (b) IL (sex*F* = 0.19711*p* = .73540; estrous*F* = 3.5331 × 10^12^,*p* = .97006), (c) vHPC (sex*F* = 3.0069,*p* = .10962; estrous*F* = 1.2988 × 10^11^,*p* = .97776), or (d) dHPC (sex*F* = 1.7919,*p* = .20312; estrous*F* = 2.1148 × 10^11^,*p* = .99954). Data represent epochs of movement 5–15 cm/s and are plotted as mean ± *SEM*; shaded error represents subjects. Female‐male comparisons were analyzed by functional two‐sample*F*‐tests; estrous stage comparisons were analyzed by functional ANOVAs. Sample sizes are found in Table 1

For each subject, LFP data from all epochs of 5–15 cm/s movement in the familiar arena were concatenated. Power spectral density (PSD) estimates were calculated from simultaneous PrL, IL, vHPC, and dHPC LFPs in the familiar arena. For each brain region, the function (shape) of broadband (0.5–100 Hz) PSDs were analyzed to contrast the biological sexes, then female data were analyzed separately as a function of estrous stage. There were no statistical differences in the function of 0.5–100 Hz PSD estimates in all four brain regions between males and females (functional two‐sample *F* tests; PrL *F* = 1.4291, *p* = .27451; IL *F* = 0.19711, *p* = .73540; vHPC *F* = 3.0069, *p* = .10962; dHPC *F* = 1.7919, *p* = .20312) or between female estrous stages (functional one‐way ANOVAs; PrL *F* = 6.6482 × 10^12^, *p* = .55955; IL *F* = 3.5331 × 10^12^, *p* = .97006; vHPC *F* = 1.2988 × 10^11^, *p* = .97776; dHPC *F* = 2.1148 × 10^11^, *p* = .99954) (Figure 2).

### 3.2 Sex‐associated difference in inter‐regional coherence without estrous modulation

**Figure 3:**
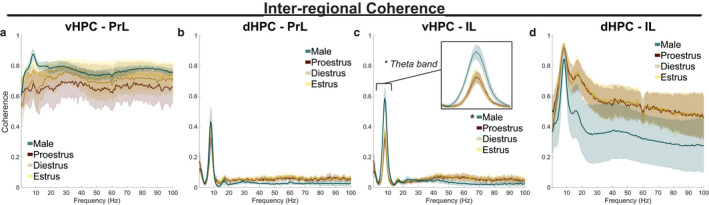
Sex‐associated difference in inter‐regional coherence. (a) The shape of 0.5–100 Hz coherence between vHPC and PrL did not statistically differ between the sexes (*F* = 0.52483,*p* = .57435) or between female estrous stages (*F* = 4.0243,*p* = .89905). (b) dHPC‐PrL coherence did not statistically differ between the sexes (*F* = 1.39414,*p* = .26884) or between female estrous stages (*F* = 0.18512,*p* = .94449). (c) While vHPC‐IL coherence was similar between female estrous stages (*F* = 0.18512,*p* = .94449), a statistical difference between males and females was observed (*F* = 3.7873, *p = .02094), an effect driven specifically by the theta band (4–12 Hz), shown in inset (*F* = 7.9277, *p = .01818). (d) dHPC‐IL coherence did not statistically differ between the sexes (*F* = 1.2212,*p* = .31942) or between female estrous stages (*F* = 0.36527,*p* = .99707). Data represent epochs of movement 5–15 cm/s and are plotted as mean ± *SEM*; shaded error represents subjects. Coherence functions were analyzed between 0.5 and 100 Hz first, then frequency bands of interest were analyzed post‐hoc. Female‐male comparisons were analyzed by functional two‐sample*F*‐tests; estrous stage comparisons were analyzed by functional ANOVAs. Sample sizes are found in Table 1

Coherence in frequency‐specific LFPs between distally connected brain regions has been theorized to play a fundamental role in coordinating their neural computations (Bastos et al., 2015). Coherence between HPC‐mPFC LFP pairs was calculated from rats moving 5–15 cm/s in a familiar arena; all epochs were concatenated by subject. For each HPC‐mPFC circuit, the functions of inter‐regional coherence between 0.5 and 100 Hz were statistically analyzed to contrast the sexes, then female data were separately analyzed to contrast the estrous stages. Coherence between vHPC and PrL did not statistically differ between the sexes (*F* = 0.52483, *p* = .57435) or between female estrous stages (*F* = 4.0243, *p* = .89905) (Figure 3a). Similarly, coherence between dHPC and PrL did not statistically differ between the sexes (*F* = 1.39414, *p* = .26884) or between female estrous stages (*F* = 0.18512, *p* = .94449) (Figure 3b). There was a statistically significant sex‐associated difference in the function of 0.5–100 Hz coherence between vHPC and IL (*F* = 3.7873, *p = .02094). This effect was driven specifically by increased theta band (4–12 Hz) coherence for males (*F* = 7.9277, *p = .01818) (Figure 3c). vHPC‐IL coherence in other frequency bands did not between the sexes (delta (1–4 Hz) *F* = 0.19999, *p* = .75107; beta (15–30 Hz) *F* = 0.37199, *p* = .66563; gamma (30–80 Hz) *F* = 1.7058, *p* = .22928)). Coherence between dHPC and IL did not statistically differ between the sexes (*F* = 1.2212, *p* = .31942) or between female estrous stages (*F* = 0.36527, *p* = .99707) (Figure 3d).

### 3.3 Hippocampal‐prefrontal theta band power correlations differ by sex and estrous stage

**Figure 4:**
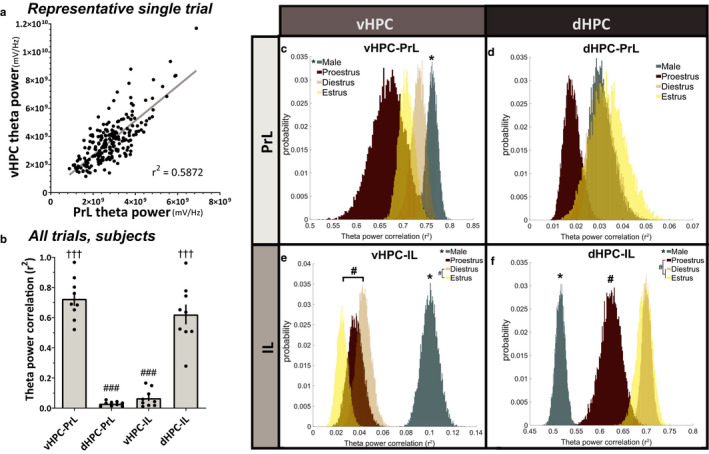
Hippocampal‐prefrontal theta band power correlations differ by sex and estrous stage. (a) Representative example of one trial's theta power correlation between vHPC and PrL in the familiar arena. Each data point represents the sum of theta band power in a 2.6s window. (b) Subject‐averaged theta power correlations between vHPC‐PrL and dHPC‐IL were significantly greater than those between dHPC‐PrL or vHPC‐IL (RM ANOVA, F_3,24_ = 76.82, ***p < .0001; Tukey's multiple comparison test,^###^ *p* < .0001 versus vHPC‐PrL,^†††^ *p* < .0001 versus dHPC‐PrL;*n* = 9 subjects). (c) vHPC‐PrL theta power correlations were significantly greater for males than females (*p = .002194). Female estrous stage did not have a statistically significant effect on vHPC‐PrL theta power correlations (diestrus/proestrus*p* = .0768; diestrus/estrus*p* = .2564; proestrus/estrus*p* = .27368). (d) dHPC‐PrL theta power correlations were not statistically different between the sexes (*p* = .6584) or between female estrous stages (diestrus/proestrus*p* = .05152; diestrus/estrus*p* = .74699; proestrus/estrus*p* = .0476). (e) vHPC‐IL theta power correlations were significantly greater for males than females (*p < 1.0 × 10^−15^). For females, estrus statistically decreased vHPC‐IL theta power correlations as compared to diestrus stage (^#^ *p* = .01471), but no other statistical differences were observed (diestrus/proestrus*p* = .43622; proestrus/estrus*p* = .17021). (f) dHPC‐IL theta power correlations were statistically greater for females than males (*p = 1.3323 × 10^−15^). While female diestrus and estrus stages were comparable (*p* = .84095), proestrus reduced dHPC‐IL theta power correlations in females (diestrus/proestrus^#^ *p* = .00115; proestrus/estrus^#^ *p* = .0081206). Data were analyzed via direct probability estimates on hierarchically‐bootstrapped samples; p_boot_values were converted to two‐tailed*p*‐values. Sample size inputs for hierarchical bootstrapping are found in Table 1

To determine how instantaneous theta band amplitudes synchronize hippocampal and prefrontal subregions, theta band power correlations between HPC‐mPFC pairs were calculated over time, and the correlation coefficients were collected per trial (Figure 4a). Comparing subject means, theta band power correlations between HPC‐mPFC circuits differed significantly from one another (Repeated‐measures ANOVA; *F*_3,24_ = 76.82, ***p < .0001, *n* = 9 subjects), such that theta power correlated more strongly in vHPC‐PrL and dHPC‐IL circuits as compared to dHPC‐PrL and vHPC‐IL circuits (Tukey's multiple comparisons test; vHPC‐PrL/dHPC‐PrL *^###^p* < .0001; vHPC‐PrL/vHPC‐IL *^###^p* < .0001; vHPC‐PrL/dHPC‐IL *p* = .3135; dHPC‐PrL/vHPC‐IL *p* = .9228; dHPC‐PrL/dHPC‐IL *^###^p* < .0001; vHPC‐IL/dHPC‐IL *^###^p* < .0001) (Figure 4b). Correlation coefficients were then analyzed as a function of biological sex and as a function of the female estrous stage using a hierarchical bootstrapping method of data resampling (Saravanan et al., 2019). Theta band power correlations between vHPC‐PrL were greater in males than females (p_boot_ = 0.001097, *p = .002194), but female estrous stage did not have a statistically notable impact (proestrus/diestrus p_boot_ = 0.03839, *p* = .07680; estrus/diestrus p_boot_ = 0.12820, *p* = .25640; estrus/proestrus p_boot_ = 0.86316, *p* = .27368) (Figure 4c). Theta band power correlations between dHPC‐PrL did not statistically differ between the sexes (p_boot_ = 0.32920, *p* = .65840) or between female estrous stages (proestrus/diestrus p_boot_ = 0.02576, *p* = .05152; estrus/diestrus p_boot_ = 0.62650, *p* = .74699; estrus/proestrus p_boot_ = 0.86316, *p* = .04760). Notably, proestrus females showed a reduction in dHPC‐PrL theta power correlations as compared to other females and males that approaches statistical significance (proestrus/males p_boot_ = 0.01195, *p* = .02391) (Figure 4d). Similarly to the vHPC‐PrL circuit, vHPC‐IL theta band power correlations were greater in males as compared to females (p_boot_ < 1.0 × 10^−15^, *p < 1.0 × 10^−15^), suggesting that theta band signaling in vHPC‐mPFC circuits is of greater amplitude in male rats as compared to females. Females in estrus displayed a decrease in vHPC‐IL theta power correlations as compared to diestrus females (estrus/diestrus p_boot_ = 0.007356, ^#^ *p* = .01471). However, vHPC‐IL theta power correlations in proestrus did not statistically differ from either diestrus or estrus (proestrus/diestrus p_boot_ = 0.21811, *p* = .43622; estrus/proestrus p_boot_ = 0.08511, *p* = .17021) (Figure 4e). Last, dHPC‐IL theta band power correlations were greater for females as compared to males (p_boot_ = 0.99999, *p = 1.3323 × 10^−15^). Proestrus stage reduced dHPC‐IL theta power correlations in females (proestrus/diestrus p_boot_ = 5.7506 × 10^−4^, ^#^ *p* = .00115; estrus/proestrus p_boot_ = 0.99594, ^#^ *p* = .0081206; estrus/diestrus p_boot_ = 0.42047, *p* = .84095), yet remained distinguishable from male values (proestrus/males p_boot_ = 0.99999, *p = 4.6800 × 10^−6^) (Figure 4f). These data suggest that in females, proestrus stage downregulates dHPC‐mPFC theta power correlations, while these signals remain constant throughout diestrus and estrus.

### 3.4 Hippocampal‐prefrontal theta phase lags differ by sex and estrous stage

**Figure 5:**
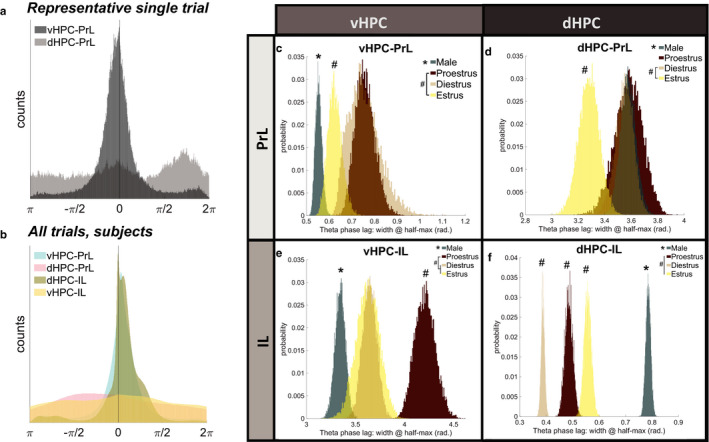
Hippocampal‐prefrontal theta phase lags differ by sex and estrous stage. (a) Representative distributions of hippocampal‐prefrontal theta phase lags from one trial: reliable theta phase synchrony from vHPC to PrL, but unreliable phase synchrony from dHPC to PrL. (b) Combining all trials, theta phase lags from vHPC to PrL (0.6193 rad.) and dHPC to IL (0.5194 rad.) were more reliably distributed than those from dHPC to PrL (5.7238 rad.) and vHPC to IL (6.2632 rad.). (c) Males had greater vHPC‐PrL theta phase synchrony (i.e. smaller width at half‐max distribution peak) than females (*p = 3.1086 × 10–15). Estrus enhanced vHPC‐PrL theta phase synchrony versus proestrus stage (*p = .01018). Diestrus female values were statistically comparable to both proestrus and estrus (diestrus/proestrus*p* = .90953; diestrus/estrus*p* = .05391). (d) Females had greater phase synchrony than males from dHPC to PrL (*p = 5.987 × 10^−4^). Female estrus trials enhanced dHPC‐PrL theta phase synchrony as compared to diestrus female trials (^#^ *p* = .010637) but were not statistically different from proestrus female trials (*p* = .026184). (e) Males had more reliable vHPC‐IL theta phase synchrony than females (*p = 2.6645 × 10^−15^). While female diestrus and estrus stages were comparable (*p* = .94046), proestrus significantly reduced vHPC‐IL theta phase synchrony versus all other groups (proestrus/diestrus^#^ *p* = 5.0000 × 10^−7^; proestrus/estrus^#^ *p* = 5.556 × 10^−5^). (f) Females had greater dHPC‐IL theta phase synchrony than males (*p < 1.0 × 10^−15^). Estrous stage statistically modulated dHPC‐IL theta synchrony in females in an order of Diestrus > Proestrus>Estrus (diestrus/proestrus^#^ *p* = 1.7764 × 10^−15^; diestrus/estrus^#^ *p* = 1.7764 × 10^−15^; proestrus/estrus^#^ *p* = 3.7229 × 10^−4^). Data were analyzed via direct probability estimates on hierarchically‐bootstrapped samples; p_boot_values were converted to two‐tailed*p*‐values. Sample size inputs for hierarchical bootstrapping are found in Table 1

Theta phase synchrony was determined by computing the distributions of theta band phase lag differences between HPC‐mPFC pairs, then comparing the bootstrap‐sampled widths at ½ the maximum distribution peaks. Calculated this way, small values indicate a sharp peak with reliable phase lags between HPC‐mPFC pairs, whereas large values indicate unreliable theta phase lag distributions between brain regions (Figure 5a). Combining all trials and subjects, theta phase lags from vHPC to PrL (0.6193 rad.) and from dHPC to IL (0.5194 rad.) were more reliably distributed (smaller width at half‐max peak values) than those from dHPC to PrL (5.7238 rad.) and vHPC to IL (6.2632 rad.), suggesting that theta phase synchrony may represent a preferential mode of communication in these circuits (vHPC‐PL and dHPC‐IL) compared to the other circuits assessed (vHPC‐IL and dHPC‐PrL) (Figure 5b). The widths at half‐maximum distribution peak for each HPC‐mPFC circuit were then analyzed as a function of biological sex and as a function of female estrous stage using a hierarchical bootstrapping method of data resampling (Saravanan et al., 2019). In the vHPC‐PrL circuit, theta phase lags were more tightly distributed in males as compared to females (p_boot_ > 0.9999, *p = 3.1086 × 10^−15^). Females in diestrus and proestrus had similar vHPC‐PrL phase lag distributions (p_boot_ = 0.54524, *p* = .90953). Estrus enhanced vHPC‐PrL theta phase synchrony versus proestrus stage (p_boot_ = 0.0005092, *p = .01018), such that estrus females were not statistically different from male values (p_boot_ = 0.98949, *p* = .02042). Estrus and diestrus females were not statistically different in vHPC‐PrL theta phase synchrony (p_boot_ = 0.026956, *p* = .05391) (Figure 5c). Conversely, theta phase lags in the dHPC to PrL circuit were more tightly distributed in females versus males overall (p_boot_ = 2.9935 × 10^−4^, *p = 5.987 × 10^−4^). In females, estrus stage increased dHPC‐PrL theta phase synchrony as compared to diestrus stage (p_boot_ = 0.0053185, *p = .010637). Females in proestrus were not statistically different from females in estrus (p_boot_ = 0.013092, *p* = .026184) or diestrus (p_boot_ = 0.62255, *p* = .75489) (Figure 5d). Between vHPC and IL, theta phase lags were more tightly distributed in males as compared to females (p_boot_ > 0.9999, *p = 2.6645 × 10^−15^). Females in diestrus and estrus were similar (p_boot_ = 0.46523, *p* = .94046), but proestrus significantly reduced vHPC‐IL theta phase lag consistency as compared to the other estrous stages (proestrus/diestrus p_boot_ = 0.99999, *p = 5.0000 × 10^−7^; proestrus/estrus p_boot_ = 2.778 × 10^−5^, *p = 5.556 × 10^−5^) (Figure 5e). Last, theta phase lag distributions from dHPC to IL were statistically different between all groups, such that diestrus females had the most reliable theta phase synchrony, followed by proestrus females, estrus females, then males (males/females p_boot_ < 1.0 × 10^−15^, *p < 1.0 × 10^−15^; proestrus/diestrus p_boot_ > 0.9999, ^#^ *p* = 1.7764 × 10^−15^; estrus/diestrus p_boot_ > 0.9999, ^#^ *p* = 1.7764 × 10^−15^; estrus/proestrus p_boot_ = 0.99981, ^#^ *p* = 3.7229 × 10^−4^). Overall, females' dHPC‐IL theta phase lags were more reliably distributed than males' (Figure 5f).

## 4 DISCUSSION

**Figure 6:**
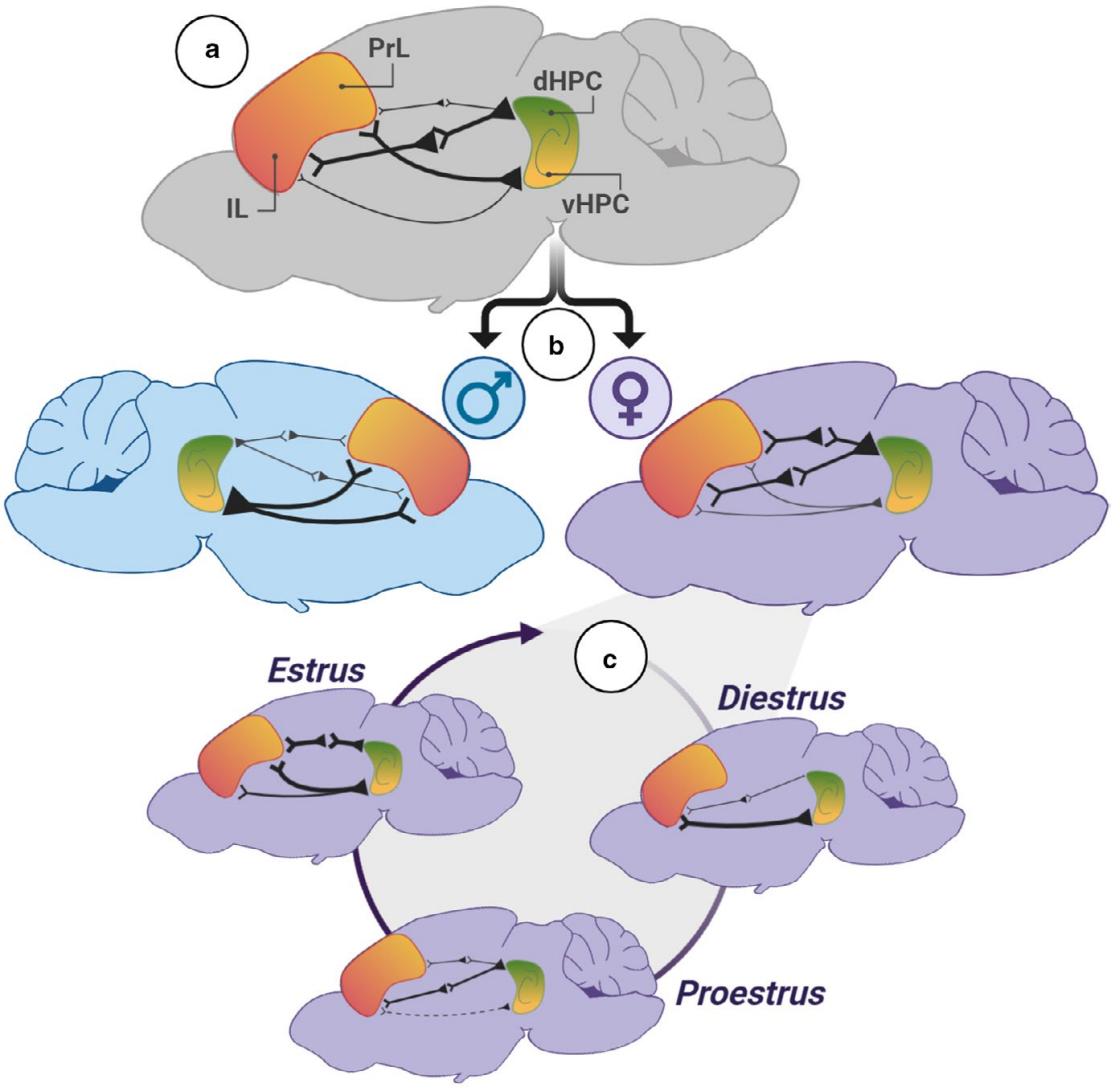
Summary of findings. (a) The strongest theta‐based circuits overall were vHPC‐PL and dHPC‐IL. (b) Sex differences in theta communications. Males had enhanced theta signaling in vHPC‐mPFC circuits as compared to females. Females had enhanced theta signaling in dHPC‐mPFC circuits as compared to males. (c) In females, estrous stage significantly affected the strength of theta‐band communications in HPC‐mPFC circuits in a dynamic fashion

Adult male and female rats were placed into a familiar arena, and LFPs from PrL, IL, vHPC, and dHPC were simultaneously recorded over many trials. Within the brain areas studied, LFP power spectral densities across a range of known brain rhythms were statistically similar between the sexes and between female estrous stages (Figure 2). While plasticity in the vHPC‐mPFC pathway has been documented (Laroche et al., 1990), coherence between HPC‐mPFC pairs in our study did not statistically differ as a function of the female estrous stage, suggesting that natural fluctuations in estradiol and progesterone alone may be insufficient to elicit observable plasticity during exploratory behaviors. Interestingly, males had greater theta band coherence than females between vHPC and IL (Figure 3c). In fact, our data suggest that males had overall greater effective theta signaling between vHPC and both mPFC subregions versus females. Males had the greatest theta power correlations between vHPC and IL/PrL (Figure 4c,e), as well as the most reliable theta phase lag distributions between vHPC and IL/PrL (Figure 5c,e). These findings align with the vHPC's known monosynaptic projections to mPFC in the male rat, whereas dHPC‐mPFC connections are believed to be polysynaptic (Hoover & Vertes, 2007). Conversely, females had greater effective theta signaling between the dHPC and mPFC subregions, though this effect appears to be stronger for IL than PrL. Proposed sex differences in HPC‐mPFC theta communications are graphically summarized in Figure 6b. It is worth noting here that the referenced neuroanatomy studies utilized male rat subjects, and literature examining the anatomy of HPC‐mPFC monosynaptic projections in females is extremely limited.

Several possible explanations for the observed male‐female differences in vHPC‐mPFC theta signaling exist. First, fewer excitatory vHPC neurons may project monosynaptically to IL and PrL in females as compared to males. Second, males may have greater vHPC theta power than females, which could drive the synchronization of vHPC‐mPFC communications differentially. However, no sex differences in vHPC power were found here (Figure 2c). Third, males could have enhanced anatomical and/or synaptic connectivity with other corticolimbic brain regions that resonate in theta with the mPFC and vHPC (for example, the basolateral amygdala). Last, there could be sex‐differential hippocampal connectivity with the medial septum or with the fimbria, believed to pace theta rhythms in male rats within dHPC and vHPC, respectively. However, additional anatomical studies are needed to fully explain the observed sex‐associated differences in vHPC‐mPFC theta connectivity.

To our knowledge, these data are the first to demonstrate several significant changes in HPC‐mPFC theta signaling across the female estrous cycle in rats. During diestrus, when circulating levels of progesterone and estradiol are low, IL theta phase preferentially synchronizes with dHPC theta phase and PL disengages, while vHPC‐IL theta power correlations are at their strongest point. During the subsequent proestrus stage, when progesterone and estradiol concentrations surge, dHPC‐IL theta phase synchrony increases at the expense of vHPC‐IL phase synchrony, and dHPC power correlations with mPFC are reduced. Finally, during the estrus stage, when circulating estradiol remains elevated but progesterone levels are diminished, PrL hits peak phase synchronization with HPC, and vHPC‐IL power correlations increase without modifying phase synchrony. Estrous‐linked changes are graphically summarized in Figure 6c. For female rats, there is likely an evolutionary benefit to having a dynamic corticolimbic system that adaptively changes the strengths of effective signaling along with the “tides” of endogenous hormone cycles. Notably, proestrus stage signals sexual receptivity in female rats (McEwen, 1981), aligning with reported increases in open‐zone exploration during approach‐avoidance conflict tasks (Frye et al., 2000; Marcondes et al., 2001; Mora et al., 1997). Therefore, one explanation may be that the hormonal surge during proestrus shapes communications within corticolimbic circuits to reduce threat bias, thereby promoting exploratory behavior and improving the odds of successfully mating. However, additional research including relevant task engagement would be required to support these claims.

Another possible explanation for the observed differences in HPC‐mPFC theta connectivity is that receptors for estrogen, progesterone, and androgens are expressed differentially between the sexes and across the female estrous stage within corticolimbic structures (Blume et al., 2017; Frye et al., 2000; Guerra‐Araiza et al., 2000, 2002; Shughrue et al., 1992; Simerly et al., 1990). Therefore, the developmental and/or activational effects of gonadal hormones (e.g., estradiol, progesterone, and testosterone) on the HPC, mPFC, and/or interconnected limbic structures may play a role in sex‐ and estrous‐related changes in theta signaling.

The vHPC‐PrL circuit has been studied in the context of approach‐avoidance behaviors to better understand its utility for identifying potential biomarkers of pro‐avoidant states, ie, modeling anxiety‐like states in rodents. Previous studies suggest that in male mice, vHPC‐PrL theta phase synchrony may serve as a functional biomarker for innate avoidance behavior in approach‐avoidance conflict tasks such as the elevated plus maze (Adhikari et al., 2010; Padilla‐Coreano et al., 2016, 2019). Extrapolating this concept, one might hypothesize that male rodents would have overall greater vHPC‐PrL theta band coupling than females, given that male rodents spend more time than females in the closed portions of the elevated plus maze when the estrous stage is ignored (Johnston & File, 1991). Our findings support the hypothesis that males have overall greater vHPC‐PrL theta band coupling than females in measurements of power correlation (Figure 4c) and phase synchronization (Figure 5c) during the exploration of a familiar arena. However, variability between subjects was too large to observe a significant effect of sex in vHPC‐PrL coherence (Figure 3c). Along with its documented role in innate avoidance behaviors, the vHPC‐PrL circuit has recently been linked with fear suppression via learned safety cues (Meyer et al.,  2019), suggesting a potential extension of these findings to learned avoidance behaviors. However, additional studies are needed to determine the role of vHPC‐PrL signaling in females in this context. While synchrony between brain regions generally tracks with anatomical connectivity at rest (Harris & Gordon, 2015), it is important to note here that the monosynaptic pathway from vHPC to mPFC is not the sole contributor to their coherence. Other inputs to the vHPC and/or the mPFC are, indeed, quite likely to modify the vHPC‐mPFC circuit's communications, depending on the cognitive state, task demand, and arousal level. A list of potential candidate brain regions worthy of additional study in this regard may include the basolateral amygdala, nucleus reuniens of the thalamus, nucleus accumbens, and the insular cortex (Vertes, 2006).

## 5 CONCLUSION

Together, data suggest that while maintaining similar power spectra within each brain region, biological sex and female estrous stage selectively modify synchronized theta frequency communications between hippocampal and prefrontal subregions during active exploration of a familiar arena. We found that theta signaling is most cohesive in vHPC‐PrL and dHPC‐IL circuits overall (Figure 6a). In male rats, vHPC‐mPFC theta signaling was more tightly coupled than dHPC‐mPFC signaling, whereas the opposite was true for females, most notably in IL (Figure 6b). In females, the estrous stage was linked with subtle yet significant shifts in theta signaling within HPC‐mPFC circuits (Figure 6c). These findings show that even in the absence of task demand, biological sex and the female estrous stage can both affect synchronized theta communications between the hippocampus and prefrontal cortex in vivo, with potential implications for biomarkers of threat representation.

## DISCLOSURE

Authors declare no conflicts of interest.

## AUTHOR CONTRIBUTIONS

KJS designed and performed experiments, conducted data analysis, performed histology, and wrote the paper. YX and WW conducted data analysis and wrote the paper. AAW assisted with data analysis, supervised experimental progress, and wrote the paper. MK designed experiments, supervised experimental progress and data analysis, and wrote the paper.
